# Continuous renal replacement therapy outcomes in acute kidney injury and end-stage renal disease: a cohort study

**DOI:** 10.1186/cc12780

**Published:** 2013-06-20

**Authors:** Andrew S Allegretti, David JR Steele, Jo Ann David-Kasdan, Ednan Bajwa, John L Niles, Ishir Bhan

**Affiliations:** 1 Department of Medicine, Massachusetts General Hospital, Boston, MA, USA; 2 Divsion of Nephrology, Department of Medicine, Massachusetts General Hospital, Boston, MA, USA; 3 Divsion of Pulmonary and Critical Care Medicine, Department of Medicine, Massachusetts General Hospital, Boston, MA, USA

**Keywords:** continuous renal replacement therapy, mortality, acute kidney injury, end stage renal disease, dialysis, dialysis-free survival

## Abstract

**Introduction:**

Continuous renal replacement therapy (CRRT) is a widely used but resource-intensive treatment. Despite its broad adoption in intensive care units (ICUs), it remains challenging to identify patients who would be most likely to achieve positive outcomes with this therapy and to provide realistic prognostic information to patients and families.

**Methods:**

We analyzed a prospective cohort of all 863 ICU patients initiated on CRRT at an academic medical center from 2008 to 2011 with either new-onset acute kidney injury (AKI) or pre-admission end-stage renal disease (ESRD). We examined in-hospital and post-discharge mortality (for all patients), as well as renal recovery (for AKI patients). We identified prognostic factors for both in-hospital and post-discharge mortality separately in patients with AKI or ESRD.

**Results:**

In-hospital mortality was 61% for AKI and 54% for ESRD. In patients with AKI (*n *= 725), independent risk factors for mortality included age over 60 (OR 1.9, 95% CI 1.3, 2.7), serum lactate over 4 mmol/L (OR 2.2, 95% CI 1.5, 3.1), serum creatinine over 3 mg/dL at time of CRRT initiation (OR 0.63, 95% CI 0.43, 0.92) and comorbid liver disease (OR 1.75, 95% CI 1.1, 2.9). Among patients with ESRD (*n *= 138), liver disease was associated with increased mortality (OR 3.4, 95% CI 1.1, 11.1) as was admission to a medical (vs surgical) ICU (OR 2.2, 95% CI 1.1, 4.7). Following discharge, advanced age became a predictor of mortality in both groups (AKI: HR 1.9, 95% CI 1.2, 3.0; ESRD: HR 4.1, 95% CI 1.5, 10.9). At the end of the study period, only 25% (*n *= 183) of patients with AKI achieved dialysis-free survival.

**Conclusions:**

Among patients initiating CRRT, risk factors for mortality differ between patients with underlying ESRD or newly acquired AKI. Long-term dialysis-free survival in AKI is low. Providers should consider these factors when assessing prognosis or appropriateness of CRRT.

## Introduction

Continuous renal replacement therapy (CRRT) is an important intervention in critically ill patients with fluid overload and metabolic disarray who are unable to tolerate the hemodynamic shifts of intermittent hemodialysis. It is expensive and resource intensive, incurring costs of up to several thousand US dollars per day [1,2]. Mortality remains high despite theoretical advances in methods and delivery over the last several years [3-6]. Identifying patients who would most achieve positive outcomes from this costly intervention is a particular challenge. In addition, the decision whether to initiate CRRT is often a critical juncture during ICU admission, as it often punctuates a time where goals of care and prognosis are revisited by care teams.

Improved assessment of prognosis is essential for guiding medical decision making and optimizing use of limited resources. Despite several attempts to identify potential risk factors for death, there is no widely accepted predictive model [7,8]. Existing analyses have primarily focused on assessing in-hospital mortality in patients with acute kidney injury (AKI) requiring CRRT [4,9-11]. In addition to AKI, however, CRRT is often employed in patients with pre-existing end-stage renal disease (ESRD) who develop hemodynamic instability. Few studies have included significant numbers of patients with ESRD who also received CRRT [3,12,13]. Furthermore, it may be useful to consider long-term outcomes in this patient population when considering the role of CRRT. We sought to address this deficit by identifying risk factors for in-hospital mortality in a cohort of patients with both AKI and ESRD who received CRRT, and continued to collect data to assess long-term outcomes following discharge.

## Materials and methods

As part of an internal quality analysis, all adult patients (age ≥20 years) who received CRRT at Massachusetts General Hospital (MGH) between 1 January 2008 and 30 September 2011 were followed prospectively after initiation of CRRT. The initial goal of this quality analysis was to track survival and renal recovery in patients requiring CRRT, to ensure they were comparable with other institutions. We used the database established as part of this process to characterize this patient population and identify prognostic factors. Patients were excluded if they remained inpatients at the end of the study period. All clinical and demographic information was reviewed daily by a registered nurse in the Division of Nephrology. Information was obtained from both paper and electronic records. Interviews with treating clinicians and patients were not performed.

MGH is a tertiary care hospital with 1,008 beds in total and 126 ICU beds available for CRRT. CRRT is provided as continuous veno-venous hemofiltration using machines from Baxter (Deerfield, IL, USA) or NxStage Medical (Lawrence, MA, USA) with pre-filter fluid replacement. Target replacement fluid rates are 20 to 30 mL/kg/hour. Bicarbonate or citrate-based replacement solutions are used. A 1:1 nursing ratio is employed for all patients on CRRT. Decisions to initiate CRRT are made by nephrologists in consultation with intensivists. At this institution, CRRT is performed in lieu of intermittent hemodialysis when patients require vasopressor support or are considered hemodynamically unstable by the consulting nephrologist.

ICU and nephrology census data and CRRT machine-usage logs were reviewed daily to ensure all patients who had initiated CRRT were included in the study. Laboratory results closest to the time of CRRT initiation were recorded from the electronic medical record, including serum blood urea nitrogen (BUN), creatinine, albumin, and lactate. Estimated glomerular filtration rate (eGFR) was calculated using the simplified Modification of Diet in Renal Disease equation [14]. BUN and creatinine from the time of admission were also captured for patients without ESRD at baseline. Demographic and clinical data were recorded, including age, gender, race, type of ICU, pre-existing diagnosis of ESRD (all of whom received chronic hemodialysis therapy prior to admission), length of stay, and Charlson comorbidity index [15]. In predictive models, continuous variables were dichotomized based on the median value to improve interpretability. For values that were available in both AKI and ESRD populations, medians for the entire population were used for dichotomization to allow for comparison between groups. Because the Charlson index score is a composite measure that incorporates a wide range of comorbidities, key components of the score were also examined as independent predictors, to determine if any particular comorbidity contributed to outcomes. Although detailed diagnostic information was not available, ICU type was dichotomized into medical versus surgical as a surrogate for the admitting diagnosis. In a randomly selected subset of 40 patients, an additional chart review was performed to further characterize differences represented by ICU type.

Patients were followed throughout the study period for assessment of recovery of renal function and death. Recovery of renal function was defined as the absence of ongoing renal replacement therapy (RRT) at the time of discharge or during the follow up period. In-hospital mortality was assessed by review of the inpatient electronic medical record. Post-discharge mortality was determined by review of the outpatient electronic medical record, public obituaries, and the Social Security Death Index.

Patients were categorized as having baseline ESRD if they had a documented history of receiving outpatient dialysis or had imminent plans to do so. For patients who had more than one admission requiring CRRT during the study period, only the first admission was considered in the analysis.

### Ethics statement

The study was approved by the institutional review board for human subjects at MGH, which waived the requirement for informed consent as the data were analyzed anonymously. All clinical investigation was conducted according to the principles expressed in the Declaration of Helsinki.

### Statistical methods

STATA 12.1 (StataCorp LP, College Station, TX, USA) was used for all statistical analysis. Univariate rank sum and chi square analyses were used for continuous and categorical variables, respectively. Factors within clinical subgroups that were statistically significant in univariate models were included in multivariate logistic regression models of survival to hospital discharge. Where both the Charlson score and one of its components were significant univariate predictors, additional multivariate models were created with only one of these two predictors, to ensure that colinearity did not mask underlying relationships. Post-discharge mortality data were assessed using Cox regression and Kaplan-Meier survival curves with log-rank testing for significance. As the goal of this study was to ascertain meaningful outcomes (for example, overall survival and dialysis-free survival) competing-risks analysis was not performed.

## Results

### General demographics

During the study period, 884 patients were initiated on CRRT. Six patients under age 20 years, who were in pediatric ICUs, were excluded. Fifteen patients (1.7% of the overall population) who underwent CRRT remained hospitalized at the end of the study period, and thus were excluded, as outcomes could not be appropriately ascertained. Excluded patients had similar baseline characteristics to those included in the final analysis. Fourteen patients had multiple admissions requiring CRRT and only the first admission was considered for these patients. After these exclusions, 863 patients remained in the analysis: 725 with AKI and 138 with ESRD. Demographic information and comorbidities for this population are outlined in Table 1. The majority of patients (67%) were between 50 and 79 years of age; 19% were younger than 50 years and 14% were 80 years or older. The distribution of ages was similar between AKI and ESRD. A majority of patients in this study (78%) self-identified as being of white race. Other reported races included black (5%), non-black Hispanic (5%), and Asian (3%). The most common comorbid factors contributing to the Charlson score included congestive heart failure (31%), diabetes mellitus (24%) and chronic pulmonary disease (20%).

**Table 1:** Baseline demographics and comorbidities

	AKI (*n *= 725)	ESRD (*n *= 138)	*P*-value
Age, years	63 (53, 74)	66 (54, 76)	0.14
Male	454 (63%)	93 (67%)	0.29
White race	564 (78%)	110 (80%)	0.62
Medical ICU	378 (52%)	93 (67%)	0.001
Lactate, mmol/L	4.3 (1.9, 8.9)	3.2 (1.5, 5.0)	0.05
Albumin, g/dL	2.8 (2.3, 3.2)	3.1 (2.6, 3.4)	0.002
BUN, mg/dL(at admission)	34 (20, 54)	N/A	N/A
Creatinine, mg/dL(at admission)	1.8 (1.2, 2.9)	N/A	N/A
BUN, mg/dL(at CRRT initiation)	54 (34, 84)	N/A	N/A
Creatinine, mg/dL(at CRRT initiation)	3.4 (2.4, 4.7)	N/A	N/A
Charlson score	2 (1, 4)	5 (4, 6)	<0.001
Length of stay, days	21 (10, 38)	19 (9, 32)	0.10
Duration of CRRT, days	5 (3, 10)	4 (3, 8)	0.10
Diabetes mellitus	208 (29%)	75 (54%)	<0.001
Chronic pulmonary disease	148 (20%)	23 (17%)	0.31
Liver disease	131 (18%)	19 (14%)	0.22
Malignancy	99 (14%)	14 (10%)	0.26
Myocardial infarction	125 (18%)	40 (29%)	0.002
Congestive heart failure	209 (29%)	57 (42%)	0.004
Peripheral vascular disease	111 (15%)	38 (28%)	<0.001
Cerebrovascular accident	91 (13%)	29 (21%)	0.008

Laboratory values used are the closest values to the initiation of CRRT, unless labeled otherwise. Values are median (interquartile range) or number (%). Medical ICU is defined as admission to a medical ICU (medical, cardiology, neurology) versus non-medical ICU (general surgery, transplant surgery, cardiothoracic surgery, burn, trauma). AKI, acute kidney injury; ESRD, end-stage renal disease; BUN, blood urea nitrogen; CRRT, continuous renal replacement therapy; NA, not assessed.

Compared with the ESRD population, patients with AKI were less likely to be admitted to a medical ICU (*P *= 0.001) and tended to have a higher serum lactate (*P *= 0.05), lower serum albumin (*P *= 0.002), and lower Charlson score (*P *<0.001). This population had a lower prevalence of diabetes mellitus (*P *<0.001), myocardial infarction (*P *= 0.002), congestive heart failure (*P *= 0.004), peripheral vascular disease (*P *<0.001), and cerebrovascular accidents (*P *= 0.008). Some laboratory values were not available for all patients, but were included when measured (*n *= 624 for serum lactate, *n *= 760 for serum albumin). Over 50% of patients with AKI had a creatinine level below 2 mg/dL on admission. Serum BUN and creatinine were not analyzed for patients with ESRD.

Of all patients, 55% were admitted to medical units: medical ICU (33%), cardiac ICU (20%), neurological ICU (2%). The remaining patients (45%) were admitted to surgical units: general surgical ICU (18%), cardiothoracic surgical ICU (23%), transplant surgical ICU (3%), and burn ICU (1%). Compared to medical ICU patients, patients admitted to the surgical ICU with AKI were older (65 versus 60 years, *P *<0.001), had higher serum lactate (7.6 versus 5.3 mmol/L, *P *<0.001) and lower serum BUN and creatinine at the time of CRRT initiation (54 versus 69 and 3.3 versus 4.2 mg/dL respectively, *P *<0.001). Fewer of them had liver disease (14% versus 22%, *P *<0.001). There was no difference in Charlson score, albumin, gender, race, or presence of diabetes. There were no differences in the surgical versus medical ICU subgroups in patients with ESRD in any of these categories. Among the 40 patients who were selected for detailed chart review, we excluded one pediatric patient who had not been included in the general analysis. Fifteen of the remaining patients had CRRT initiated in a medical ICU. Nearly all patients (88%) in surgical ICUs had an operation during the admission prior to initiating CRRT; only 13% of medical ICU patients had preceding surgery. Compared to medical ICU patients, fewer surgical ICU patients had septic shock (33% versus 17%); surgical ICU patients also had similar rates of cardiogenic shock (42% versus 40%) and lower incidence of preceding cardiac arrest (33% versus 12%). In this small sample, only the difference in rate of surgery was statistically significant.

### In-hospital mortality

Overall in-hospital mortality for patients with AKI was 60.7% compared with 54.3% in patients with ESRD (*P *= 0.16). Among patients with AKI, significant predictors included age over 60 years, lactate greater than 4 mmol/L, Charlson score over 3, and an underlying diagnosis of liver disease. Creatinine greater than 3 mg/dL at the time of CRRT initiation was associated with decreased mortality (Table 2a). Similarly, lower eGFR at the time of CRRT initiation was associated with decreased mortality (*P *= 0.004). Among patients with ESRD, admission to a medical (versus surgical) ICU, Charlson score over 3, and the presence of liver disease were predictive of mortality (Table 2b).

**Table 2:** Factors associated with in-hospital mortality among patients with acute kidney injury or end-stage renal disease

	Odds ratio	95% CI	*P*-value
**A: Acute kidney injury**			
Age >60 years	1.6	1.2, 2.2	0.002
Female	0.96	0.71, 1.3	0.82
White race	0.87	0.60, 1.2	0.43
Medical ICU	1.2	0.9, 1.6	0.19
Lactate >4 mmol/L	2.4	1.7, 3.3	<0.001
Albumin >3 g/dL	0.83	0.61, 1.1	0.26
BUN > 30 mg/dL (at admission)	1.1	0.81, 1.5	0.54
Creatinine >2 mg/dL (at admission)	0.76	0.56, 1.0	0.08
BUN >50 mg/dL (at CRRT initiation)	1.3	0.94, 1.7	0.12
Creatinine >3 mg/dL (at CRRT initiation)	0.56	0.41, 0.77	<0.001
Charlson score >3	1.6	1.1, 2.3	0.006
Liver disease	1.8	1.2, 2.8	0.005
			
**B: ESRD**			
Age >60 years	0.88	0.45, 1.7	0.72
Female	1.2	0.60, 2.5	0.57
White race	0.60	0.25, 1.4	0.24
Medical ICU	2.4	1.1, 4.9	0.02
Lactate >4 mmol/L	1.5	0.28, 7.6	0.65
Albumin >3 g/dL	0.79	0.24, 2.6	0.70
Charlson score >3	2.4	1.0, 5.7	0.05
Liver disease	3.7	1.2, 11.8	0.03

(**A**) Acute kidney injury (AKI) (*n *= 725). (**B**) End-stage renal disease (ESRD) (*n *= 138). BUN, blood urea nitrogen; CRRT, continuous renal replacement therapy.

Multivariate logistic regression modeling for in-hospital mortality was performed using variables that were significant in univariate analyses and are summarized in Table 3. In patients with AKI, all factors significant in univariate analyses continued to be predictive of mortality with the exception of Charlson score over 3. In patients with ESRD, both Charlson score and liver disease lost significance in a multivariable model. Given the possibility that this was due to colinearity between these variables, we generated multivariable models with only one of these two predictors along with ICU type. As liver disease was a stronger predictor of mortality than Charlson score over 3, this was included in the final multivariable model (Table 3b).

**Table 3:** Multivariable models identifying factors predictive of in-hospital mortality

	Odds ratio	95% CI	*P*-value
**A: AKI**			
Age >60 years	1.9	1.3, 2.7	0.001
Lactate >4 mmol/L	2.2	1.5, 3.1	<0.001
Creatinine >3 mg/dL (at CRRT initiation)	0.63	0.43, 0.92	0.01
Charlson score >3	1.4	0.92, 2.1	0.12
Liver disease	1.75	1.1, 2.9	0.03
			
**B: End-stage renal disease**			
Medical ICU	2.2	1.1, 4.7	0.03
Liver disease	3.4	1.1, 11.1	0.04

(**A**) Acute kidney injury (AKI) (*n *= 725). (**B**) End-stage renal disease (ESRD) (*n *= 138). CRRT, continuous renal replacement therapy.

### Post discharge follow-up

**Figure 1:**
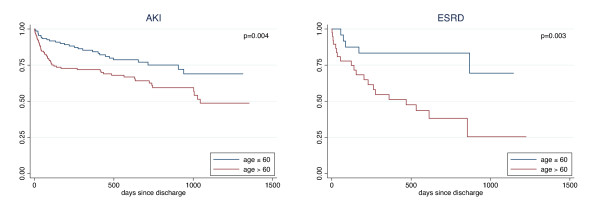
**Kaplan-Meier curves for survival following discharge, by age**. Advanced age predicted mortality in patients with either acute kidney injury (AKI; *n *= 285) or baseline end-stage renal disease (ESRD; *n *= 63).

A total of 347 patients who survived to hospital discharge were followed until the end of the study period, with a median follow up of 440 days (IQR 109 to 759). Among these patients, 28.4% (*n *= 81 out of 285) of patients with AKI and 39.7% (*n *= 25 out of 63) of those with ESRD died during the follow up period. Age over 60 years was a significant predictor of post-discharge mortality in both AKI (hazard ratio (HR) 1.9, 95% CI 1.2, 3.0) and ESRD (HR 4.1, 95% CI 1.5, 10.9); Kaplan-Meier survival curves for age are presented in Figure 1. Additional risk factors for patients with AKI included BUN over 30 mg/dL on admission (HR 2.1, 95% CI 1.3, 3.4), BUN at CRRT initiation over 50 mg/dL (HR 2.0, 95% CI 1.3, 3.2), and Charlson score over 3 (HR 2.0, 95% CI 1.3, 3.2).

Among those with AKI who survived until discharge, 89% (*n *= 255) were no longer dialysis-dependent by hospital discharge. An additional nine patients eventually recovered renal function by the end of the study period, resulting in a total rate of renal recovery of 93%. No demographic or laboratory data significantly predicted renal recovery in this subpopulation (see Table 4). In total, only 25% of patients with AKI (*n *= 183) achieved dialysis-free survival by the end of the follow up period (median follow up 449 days).

**Table 4:** Odds of requiring ongoing dialysis at discharge among surviving patients with acute kidney injury (*n *= 285)

	Odds ratio	95% CI	*P*-value
Age >60 years	1.8	0.81, 3.9	0.15

Female	0.80	0.36, 1.8	0.59

White race	1.3	0.49, 3.7	0.57

Medical ICU	1.6	0.76, 3.5	0.21

Lactate >4 mmol/L	0.91	0.32, 2.6	0.85

Albumin >3 g/dL	0.82	0.36, 1.9	0.64

BUN > 30 mg/dL (at admission)	1.7	0.76, 3.8	0.20

Creatinine >2 mg/dL (at CRRT admission)	1.3	0.63, 2.9	0.44

BUN >50 mg/dL (at CRRT initiation)	1.7	0.78, 3.7	0.18

Creatinine >3 mg/dL (at CRRT initiation)	2.1	0.83, 5.3	0.12

Charlson score >3	1.5	0.65, 3.4	0.35

BUN, blood urea nitrogen; CRRT, continuous renal replacement therapy.

## Discussion

Because of the lack of conclusive data surrounding outcomes of patients with either AKI or ESRD who receive CRRT, we analyzed a prospective cohort of all patients receiving CRRT at a major academic medical center. We found that mortality rates were similar between patients with AKI or ESRD, at 61% and 54%, respectively. We additionally found that 28% of surviving patients with AKI and 40% of those with ESRD died in the period following discharge, suggesting that in-hospital mortality rates may underrepresent the impact of CRRT-associated disease on survival.

In addition to questions about the likelihood of survival, patients and their families often inquire about the likelihood of renal recovery when assessing escalation of care, including CRRT. We found that the rate of renal recovery among patients with AKI who survived to discharge appeared at first glance to be high (89% at discharge, improving to 93% by the end of the study period), similar to previously reported rates [9,16]. However, it may be more appropriate to frame this information as dialysis-free survival; only 25% of all patients with AKI who required CRRT remained both alive and off dialysis by the end of the study period. Our study was not intended to dictate the appropriateness of offering CRRT to a patient with AKI, but rather to help clinicians frame their goals of care and prognostic discussions in the most realistic terms.

When examining individual potential predictors of mortality, we found that risk was modified by several baseline factors. Furthermore, we found that these risk factors differed based on whether patients had pre-admission ESRD, or developed new AKI during the admission. Whereas other studies have examined both groups, prior analyses have included smaller numbers of patients and a wide range of severity of AKI, rather than an entire population of patients requiring CRRT [12,13]. To our knowledge, this is the largest study to exclusively examine a CRRT cohort of patients with AKI and ESRD in parallel. Of particular note, patients with AKI and ESRD appear to have similar rates of in-hospital mortality. Depending on the clinical scenario, there may be a presumption that patients with ESRD are inherently sicker than those with AKI, and thus would be worse candidates for CRRT. Our results refute this assertion. The results also emphasize that these subgroups have distinct risk factors for mortality.

Among patients in our study with AKI, age over 60 years, serum lactate greater than 4 mmol/L at initiation of CRRT, and comorbid liver disease were independent risk factors for mortality. Age may be an indirect baseline measure of health and ability to recover from critical illness. Other studies have also identified age as a risk factor for in-hospital death in severe AKI [9-11,16,17]. The association of liver disease with the outcomes may reflect the high mortality associated with hepatorenal syndrome [18]. An elevated lactate level likely reflects the severity of the underlying insult, and thus portends a worse outcome. Interestingly, a creatinine value greater than 3 mg/dL at initiation of CRRT was associated with lower mortality. This may reflect the fact that creatinine values represent underlying muscle mass in addition to renal function. While eGFR is not a reliable measure of kidney function in AKI, we used it to reduce the potential confounding effects of age, race, and gender on creatinine generation. Analogous to creatinine, we found that lower eGFR was associated with decreased mortality. Others have suggested that poor baseline renal function in a non-ESRD population may actually be protective [19,20], supporting the concept that patients with advanced kidney disease require a less severe insult to necessitate transition to CRRT. Additionally, fluid balance may influence creatinine values in critically ill patients [21]. The association of higher creatinine with improved outcomes in AKI is intriguing, but requires further investigation to understand its true implications. Surrogate measures of muscle mass were not collected as part of the routine clinical care of this cohort, but future studies could explore this association more directly. Lastly, scores for the assessment of the severity of illness, such as the acute physiology and chronic health evaluation (APACHE) II or sequential organ failure assessment (SOFA) score were not used clinically and thus, were unavailable in our analysis. These could be studied in prospective analyses that calculate these scores.

Predictors of mortality in ESRD were strikingly different from those in AKI. This may reflect the different pathways by which these two populations come to be treated with CRRT. A patient with ESRD has a baseline requirement for RRT and thus, only requires a superimposed hemodynamic insult in order to require CRRT. Relatively mild perioperative hypotension in the absence of severe illness might be adequate to cause a patient with ESRD to end up on CRRT in a surgical ICU. In contrast, a medical ICU admission would more likely to be associated with a significant systemic insult (for example, septic shock). A patient with AKI, on the other hand, requires an insult sufficient to cause both kidney injury and hemodynamic compromise. A recent review examining ICU admissions among patients with ESRD showed that these patients may have favorable short- and long-term outcomes compared to those with AKI [22]. Our data support the conclusion that these groups should be viewed distinctly, and that our current illness severity scores may not be equally valid in both populations.

In the general ICU population, long-term post-discharge survival has been well described. One-year mortality rates in those who survive to ICU discharge have been reported between 5 and 15% [23,24], with certain disease processes, such as ARDS and sepsis, conveying poorer long term prognoses [25-29]. In AKI, few studies have examined the long-term effects of illnesses requiring CRRT. Those that have are limited by having only one year of follow up or less, and have included inconsistent patient subgroups, such as exclusively surgical patients [30,31], patients on either CRRT or intermittent hemodialysis [16,20,31], and severe AKI that did not necessarily require renal replacement [32]. Similarly, there are few studies of long-term outcomes in ESRD, but these have not focused on patients receiving CRRT [3,32-34]. Prior literature echoes some of our findings in AKI, identifying long-term predictors of mortality that include age [16,30-32], high Charlson score [16,20], and medical diagnosis [32]. We also found that elevated BUN, both at admission and initiation of CRRT, were predictive of post-discharge mortality, in contrast to the protective association of an elevated creatinine in the inpatient settings. This may reflect the unique association of creatinine with muscle mass. Interestingly, in ESRD, age was a predictor of post-discharge mortality, but not inpatient mortality. This may reflect the broad range of disease severity that leads to initiation of CRRT in the hospital, while baseline health (as reflected by age) is more important in the outpatient setting. Indeed, prior literature suggests that in patients with ESRD, the risk of mortality may return to the pre-ICU admission baseline risk within 6 months of discharge [22,32-34].

By its observational design, we were limited by the laboratory data available to the treating clinicians for each patient. As a result, we could not investigate potential novel predictors of mortality. Volume overload has been associated with worse in-hospital and post-discharge outcomes, but was not quantified in our dataset [35-37]. Additional factors, such as urine output, chronic kidney disease (CKD) stage among patients in the AKI group pre-hospitalization, reason for CRRT initiation, and dialysis vintage in the ESRD group, could be captured prospectively in future studies. Efficacy measures for CRRT, such as metabolic control and downtime could also clarify potential mediators or effect modifiers.

Because there were no coded admitting diagnoses available in this database, we used admission to medical versus surgical ICU as a proxy for underlying disease process. This yields both limitations and benefits to our conclusions: the admitting diagnosis is often subjective, especially in complex ICU patients with multi-system organ dysfunction, as it is not always possible to determine if one disease process is the culprit for renal failure. Given our database size, it is unlikely that individual diagnoses would have obtained sufficient power to provide statistically significant results. In a detailed review of medical records from a subset of patients in this study, we unsurprisingly found that patients in surgical ICUs were considerably more likely to be postoperative and somewhat less likely to have septic shock.

Admission to a medical ICU predicted in-hospital mortality only in patients with ESRD. We found no differences between surgical and medical ICU patients in age, race, gender, Charlson score, serum albumin, or serum lactate, making it unlikely that these factors confounded the relationship between ICU type and mortality.

As both the decision regarding the timing of the initiation and the type of RRT is subjective and affected by both resources and underlying disease, this single-center study may not be completely generalizable to sites with different practice patterns or patient populations. However, most of the key demographics for this population, such as age and race, were consistent with prior reports [9,32]. Because of the small number of patients who survived to discharge, we had less power to identify predictors of renal recovery and mortality post discharge. Given the observational design that precluded direct confirmation of patient mortality, it is possible that we underestimated post-discharge mortality. However, in contrast to other studies where follow up was more restricted, we were able to provide nearly 4 years of rolling post-discharge data.

## Conclusions

Patients undergoing CRRT with AKI and ESRD have different risk factors for mortality, both in the short and long term. Mortality is high in both groups, even among patients who survive to hospital discharge. Patients with ESRD should not be inherently considered poor candidates for CRRT when compared to those with AKI. The vast majority of patients with AKI who receive CRRT will not achieve long-term dialysis-free survival. Clinicians should be aware of these statistics (for example, 25% likelihood of dialysis-free survival in AKI patients started on CRRT) when counseling patients and their families. Further studies could assess the impact of this information on medical decision making, validate the results of this study in additional centers, and assess health-related quality of life among survivors.

## Key Messages

• In-hospital mortality is approximately 60%, both for patients with AKI and ESRD

• Predictors of mortality differ between these groups, likely reflecting differences in the clinical processes that lead to CRRT initiation

• While the frequency of renal recovery among survivors with AKI exceeds 89%, high rates of mortality before and after discharge result in an overall dialysis-free survival rate of only 25% (with a median follow up of 449 days)

## Abbreviations

AKI: acute kidney injury; APACHE: acute physiology and chronic health evaluation; BUN: blood urea nitrogen; CKD: chronic kidney disease; CRRT: continuous renal replacement therapy; eGFR: estimated glomerular filtration rate; ESRD: end-stage renal disease; HR: hazard ratio; IQR: interquartile range; MGH: Massachusetts General Hospital; OR: odds ratio; RRT: renal replacement therapy; SOFA: sequential organ failure assessment.

## Competing interests

The authors declare they have no competing interests.

## Authors' contributions

AA drafted the manuscript and participated in statistical analysis. DS and JN contributed to the study design and coordination of the study. JDK maintained the patient database and assisted in data analysis. EB helped draft the manuscript and assisted in data analysis. IB helped draft the manuscript and performed statistical analysis. All authors read and approved the final manuscript.

## Authors' information

AA is a senior resident in internal medicine at MGH. DS, JN, and IB are nephrologists at MGH, active in clinical research. JDK is a research nurse. EB is the Director of the Medical Intensive Care Unit at MGH.
